# Augmented reality for chemistry education to promote the use of chemical terminology in teacher trainings

**DOI:** 10.3389/fpsyg.2022.1037400

**Published:** 2022-11-23

**Authors:** Melanie Ripsam, Claudia Nerdel

**Affiliations:** Associate Professorship of Life Sciences Education, TUM School of Social Sciences and Technology, Technical University of Munich, Munich, Germany

**Keywords:** augmented reality, AR learning environment, chemical terminology, (multiple) external representations, representation change, substance-particle concept understanding, teacher education and training, split attention

## Abstract

Chemistry as a whole is divided into three levels. The *macroscopic level* describes real, observable phenomena of the material world. The *submicroscopic level* focuses on particles. The *representative level* includes pictorial and symbolic representations to visualize substance in its nature. Students often have problems separating these levels and conceptually transfer each of the three levels to the other. Therefore, teachers need to use chemical terminology correctly when teaching the substance-particle concept. Augmented reality (AR) connects the real and virtual worlds. The observer physically moves in a real environment that integrates virtual elements. This can be effective for learning when chemical processes that are invisible are made visible. The simultaneous presentation should avoid split attention and offers new possibilities to interactively deal with multiple external representations ((M)ER). The question arises whether AR has a positive effect on the use of technical language. With an AR app on the tablet and on the hololens, chemical processes of a real experiment are represented by AR visualizations. In this study, the chemistry terminology of chemistry teachers (*N* = 30) was captured using a pre-post survey. Each test includes five tasks elaborated by thinking aloud. Therefore, the AR app was piloted. The thinking-aloud protocols to acquire the use of the chemical terminology are evaluated in MAXQDA.

## Introduction

According to Johnstone (2000), chemistry as a whole is divided into three levels: (1) The Macroscopic Level describes real, observable phenomena of the material world. (2) The Submicroscopic Level focuses on particles such as atoms, ions, molecules, and chemical processes. (3) The Representative Level includes pictorial and symbolic representations (such as texts, symbols or images) to visualize substance in its nature macroscopically or submicroscopically. If learners can conceptually transfer each of the three levels to the other, this should have a positive effect on the learning process (cf. Devetak et al., 2004; Farida et al., 2010). International studies show that students use the particle concept inconsistently and that chemical terminology, with its multiple external representations ((M)ER), is very challenging (Harrison and Treagust, 2000). However, it also seems difficult for chemistry teachers to learn and teach three-level thinking (Justi and Gilbert, 2002; van Driel and Verloop, 2002). For example, the change from substance to particle level is not sufficiently emphasized in lesson design. Also the language skills are often deficient (Rodić et al., 2018). It makes sense to integrate digital media as a supporting measure in subject teacher training (Sailer et al., 2017). A benefit should arise from technological advances when visually imperceptible processes are made visible with digital software systems (Farida et al., 2010). Particle modeling techniques (e.g., tablet with video) contribute to substance-particle concept understanding (Schnitker, 2016). However, in such settings, the viewer is forced to look back and forth between the medium and the real experimental setup. The split attention effect can disrupt cognitive processing during text-image integration (Schnotz and Bannert, 2003; Ayres and Sweller, 2014). Augmented reality (AR) links real and virtual worlds (Ibanez and Delgado-Kloos, 2018) so that the observer physically moves in a real environment that integrates virtual elements. In this way, AR enables interaction with real and virtual objects (Azuma, 1997). When submicroscopic particles are virtually superimposed on the experiment (while a real experiment is running), the information can be spatially and temporally connected as well as semantically linked (Chavan, 2016). In addition to visualizing particles such as electrons, virtual overlays in a real experiment environment can also consist of chemical symbols (reaction equations) or texts such as (technical terms) and should be used in a supportive manner depending on the prior knowledge of the viewer (Schnitker, 2016; Akçayır and Akçayır, 2017; Nerdel, 2017). In this way, cognitive processing can be controlled in a self-regulated way. According to the coherence and contiguity principle of Mayer (2014), this simultaneous presentation should avoid split attention and offers new opportunities for successful learning in the levels according to Johnstone (2000).

### Aim and scientific questions

This study focuses on the learning effectiveness of an AR learning environment (on tablet or hololens) to promote the use of chemical terminology, i.e., dealing with (M)ER. Therefore, a learning environment was designed that is aimed at teachers to expand their professional knowledge. The target is to be able to use innovative digital technologies in the subject lessons with students in a perspective and didactically reflected way. The following questions will be investigated in the research project:

(1) Can the AR learning environment be used to promote reflective use of technical language at the substance and particle level from a teaching perspective among chemistry teachers?

It is hypothesized that the use of AR learning environment promotes the integration of the representation level when observing a real experiment and improves in this context the substance-particle concept understanding.

(2) Can the interactive use of augmented representational forms in the learning environment, with regard to the use of tablet or hololens, describe different elaboration profiles?

It is hypothesized that the use of the AR learning environment on a tablet has a positive effect on the use of chemical terminology. The simultaneous linking of AR representations with the content of the real experimental environment is expected to initiate cognitive processing. In comparison, the use of the simulation should disrupt cognitive processing and improve chemical terminology to a much lesser extent. By avoiding split attention, AR is expected to support the construction of mental models and thus largely shape elaboration behavior.

Besides this, (M)ER can be controlled in a self-regulated way. Different elaboration profiles are expected when interacting with augmented (M)ER on the tablet *or* hololens.

## Methods

### Participants

The subjects are teachers from German secondary schools who teach chemistry (*N* = 30). Experimental group 1 consists of 10 subjects working with AR learning environment on the tablet. Furthermore, experimental group 2 works with the same AR learning environment on a hololens. The control group consists of 10 other subjects working with a content equivalent simulation-based learning environment on the tablet.

### Design of the augmented reality learning environment

**FIGURE 1:**
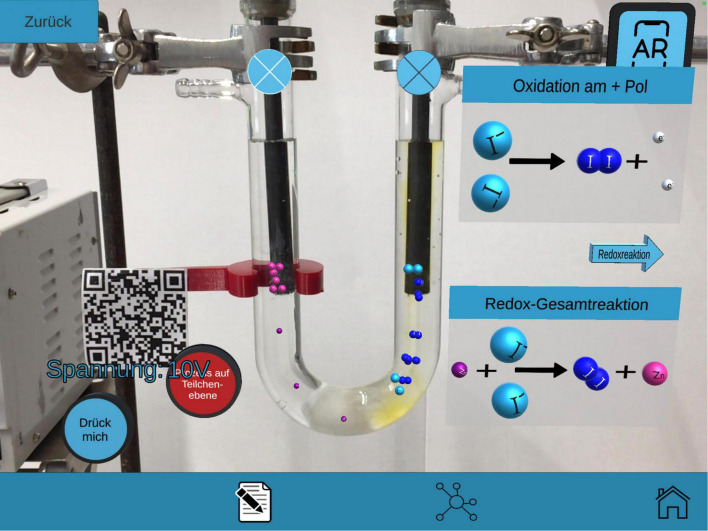
View through a tablet on the real experiment with virtual overlays of the learning path *chemical reaction*: Pictorial representation after clicking the buttons “Oxidation” and “Redox reaction” with the particle level processes (after switching on the DC voltage source).

The AR learning environment on the topic of redox reactions consists of a real experimental setup for the electrolysis of zinc iodide. As soon as the subjects point to a tablet/look through a hololens with the app at the electrolysis cell, the virtual learning environment appears in the foreground (Chavan, 2016). The function menu can be used to interactively direct which (M)ER are virtually projected onto the real experiment (Schmalstieg and Höllerer, 2016). The AR setting includes four learning paths that are elaborated before and after the DC source is turned on: *Experimental Setup*, *Diffusion* and *Electrolysis at the Particle Level*, *Chemical Reactions*. Within a learning path, concrete changes in the presentation were integrated in terms of content: The user has the possibility to distinguish between the presentation forms *text*, *symbol*, and *image*. Figure 1 exemplifies that the user can view the chemical reactions pictorially and simultaneously project the particle-level processes into the real experiment. The particle-level processes are always oriented to the real experiment sequence at the substance level (e.g., Azuma, 1997).

With the aim of adapting the contents of the AR learning environment to the needs and prior knowledge of the learners, a manageable set of ions was chosen. Cognitive load (cf. Sweller et al., 1990) was thus to be avoided. In particle modeling, attention was paid to ion size ratios and atomic and molecular radii, but their diameters or radii were not specified numerically. Since both electrolysis and diffusion are already two significant, extensive chemical topics, dissociation was not directly integrated. A help button can be clicked to get information about the hydrate sleeves.

### Survey instruments and method

#### Questionnaire

The content of the pre-test and post-test is adapted to record the use of chemical terminology. Each test includes five tasks that capture the handling of (M)ER. The tasks refer to the donor-acceptor principle. They always focus on the construction, interpretation, and translation of (M)ER. In order to analyze the effect of AR on technical language and the related construction of mental models about redox reactions, the method of thinking aloud is used. For this purpose, subjects’ utterances are recorded during the processing of the tasks and the AR learning environment.

#### Data collection

In order to investigate technical language via the elaboration behavior of virtual representations in the AR environment among chemistry teachers (see chap. “Aim and scientific questions”; Research Questions 1 and 2), all subjects in the study participate in a pre-post survey: Before starting the learning environments, subjects are informed about what they need to pay attention to when completing the tests and thinking aloud. This is followed by the completion of the pre-test. Teachers are then briefly instructed on how to use the digital device (AR or simulation-based technology) on the tablet/hololens. Afterward, the experimental group 1 resp. 2 works on the AR learning environment on the tablet resp. hololens and the control group on the simulation-based learning environment. The simulation-based learning environment is designed to be similar in content to the AR environment but has—compared to the AR app—on the tablet a detrimental split attention effect from a cognitive psychology perspective (Azuma, 1997; Mayer, 2014; Schnitker, 2016). During the interaction with the AR-App or simulation, subjects are asked to describe the experiment thinking aloud and to explain the process at the particle level with (M)ER (cf. chap. “Design of the AR learning environment”). The post-test to assess the understanding of the technical language concludes the data collection (see Research Questions in chap. “Aim and scientific questions”).

#### Analysis methods

The thinking-aloud protocols of processing the test tasks will be analyzed with qualitative content analysis according to Mayring (2010) until the category system is fully validated. Therefore, the statements will be transcribed (Bortz and Döring, 2006). The categorization and coding of the transcripts will be done with MAXQDA. For the qualitative analysis, a category system based on Kroß and Lind (2001) will be used. The category system is based on five main categories, which always differentiate between text, symbol and image. In this context, mainly inferences (e.g., building a situation model) should be recorded. This will capture whether types (Mayring, 2010) emerge regarding elaboration in the AR (un)supported learning environment. After deductive category formation, the category system will be inductively finalized by analyzing the data material. 20–25% of the data material is double-coded by two independent raters to assess the appropriateness of the categorization (Bortz and Döring, 2006). Quantitative coding of the (main) categories (Wirtz, 2013) aggregates the data. By determining frequencies of individual trait expressions, trait profiles of the subjects will be obtained (see chap. “Aim and scientific questions”).

## Pilot study on the evaluation of the augmented reality learning environment

If AR is to be applied in the classroom, the teaching and learning offer must be accepted by the teachers (Bürg, 2005). Acceptance requires a positive assessment of the information/system quality of the innovation (content and characteristics of the learning environment/usability) by the target group (Figl, 2010). Therefore, the pilot study examined, how science experts evaluate the features of the AR learning environment (usability) and to what extend they accept the learning environment.

### Participants

In March 2021, the review of the AR learning environment (acceptance/usability) took place (*N* = 18). Natural scientists, (prospective) chemistry teachers, science educators, and software developers were interviewed, all of whom use digital media regularly. Half of the subjects consisted of teachers.

### Materials

The task was to pilot the beta version of the AR learning environment. This was the setting described conceptually in chapter “Design of the AR learning environment.” At the time of piloting, it was a simplified layout with navigation through the learning paths, which was not intuitive enough. Furthermore, the programming of the perspective changes (e.g., change position of the tablet/zoom into the U-tube) had not been completed. Help buttons were missing and particle modeling was undeveloped.

### Procedure

All subjects processed the AR learning environment using a tablet. During the interaction with the AR learning environment, the participants had to explain the processes on the particle level with different representations. Subsequently, the questionnaire on the acceptance and usability of the AR learning environment was completed by the subjects.

### Questionnaire

During the piloting of the AR learning environment, scales according to Kopp et al. (2003) on acceptance, assessment of didactic and media-didactic design criteria, technical facilitation of learning, learning process, and anticipated learning success/learning transfer are used to investigate the suitability of the AR learning environment against the backdrop of research questions 1 and 2 (see chap. “Aim and scientific questions”). Questionnaire development was also based on prior work by ISO 9241-11 (1998), Bürg (2005), and Wolf and Söbke (2020).

### Results

A reliability analysis of the AR learning environment provided predominantly good to excellent internal consistency values:

The Acceptance scale (example item: “*I would use the AR learning environment in my own chemistry classes.*”) with a total of 7 items has a Cronbach’s alpha of 0.73. The eight Usability scales (example item: “*The AR learning environment is likely to spark learners’ curiosity about redox reactions at the material and particle levels.*”) with 4–17 items per scale also show a Cronbach’s alpha between 0.668 and 0.904. Furthermore, all scales on the characteristics of the learning environment have mean values above the mean scale level (see Table 1).

**TABLE 1:** Descriptive statistics and quality of the nine scales from questionnaire on a four-point Likert scale from 0 = I do not agree to 3 = I agree completely; number of items (N), mean values (M), standard deviation (SD), and Cronbach’s alpha (α) are given.

Scales	*N*	*M*	*SD*	α
Acceptance	7	2.35	2.99	0.737
Instructional support	6	1.88	2.91	0.668
Technical usability	9	2.19	4.40	0.764
Individualization	4	2.35	1.88	0.669
Problem-oriented didactics	17	2.32	6.49	0.885
Comprehensibility of media	12	2.48	5.11	0.827
Media effect	15	2.38	5.84	0.828
Learning process: anticipated motivation	6	2.31	2.95	0.874
Learning process: expected learning success	17	2.20	8.36	0.904

### Discussion and outlook

In our pilot study, the conception of the learning environment, despite small flaws, is rated very positively. This positive assessment of usability provides first indications that the setting is accepted by the subjects. Based on the pilot results, the AR learning environment and test instruments were optimized (cf. final version in section “Design of the AR learning environment”) to be used in the main study.

## Data availability statement

The original contributions presented in this study are included in the article/supplementary material, further inquiries can be directed to the corresponding author.

## Ethics statement

The studies involving human participants were reviewed and approved by the Ethics Committee of the Technical University of Munich. The patients/participants provided their written informed consent to participate in this study.

## Author contributions

MR was a Ph. D. student under the supervision of CN at TUM. In the context of the doctoral project, the presented study was carried out. Both authors contributed to the article and approved the submitted version.
